# Molecular analysis of integrons and antimicrobial resistance profile in Shigella spp. isolated from acute pediatric diarrhea patients

**DOI:** 10.3205/dgkh000308

**Published:** 2018-01-31

**Authors:** Mohammad Mehdi Soltan Dallal, Sajjad Omidi, Masoumeh Douraghi, Mohammad Taghi Haghi Ashtiani, Mohammad Kazem Sharifi Yazdi, Arash Okazi

**Affiliations:** 1 Department of Food Microbiology, School of Public Health, Tehran University of Medical Sciences, Tehran, Iran; 2 Food Microbiology Research Center, Tehran University of Medical Sciences, Tehran, Iran; 3 Division of Microbiology, Department of Pathobiology, School of Public Health, Tehran University of Medical Sciences, Tehran, Iran; 4 Division of Pathology, Children’s Medical Center, Tehran University of Medical Sciences, Tehran, Iran; 5 Zoonosis Research Center, Tehran University of Medical Sciences, Tehran, Iran; 6 Department of Medical Laboratory Sciences, School of Para Medicine, Tehran University of Medical Sciences, Tehran, Iran; 7 Forensic Medicine, School of Medicine, Tehran University of Medical Sciences, Tehran, Iran

**Keywords:** integrons, Shigella spp., acute pediatric diarrhea, multiplex PCR

## Abstract

**Introduction:** *Shigella* spp. is a growing global health concern due to increasing multiple drug resistance, commonly resulting in therapeutic failure. Integrons are gene expression systems run by integrase genes. The aims of this study were detection of class I, II and III integrons and assessment of antimicrobial resistance in *Shigella* spp. isolated from acute pediatric diarrhea patients.

**Materials and methods:** From January to December 2015, 16 *Shigella* spp. were isolated from 310 non-duplicative diarrheal stool samples in Children’s Medical Center, Tehran, Iran. The isolates were analyzed for their antibiotic susceptibility using CLSI guidelines M100-S14. Multiplex PCR was used for amplification of I, II and III integron-associated integrase (*intl*) genes.

**Results:** Of 310 stool samples, 16 (5.2%) were positive for *Shigella* spp., in 7 of them *S. sonnei* and in 9 of them *S. flexneri* were identified. Results of the antimicrobial susceptibility test showed that 6.2%, 50%, 31.2%, 6.2%, 81.2%, 56.2% and 31.2% of the isolates were resistant to gentamicin, chloramphenicol, nalidixic acid, ciprofloxacin, tetracycline, ampicillin and trimethoprim-sulfamethoxazole, respectively. Multiplex PCR results revealed that 6.2% (1/16), 31.2% (5/16), 50% (8/16) of *Shigella* isolates carried *intl*I, i*ntl*II and both *intl*I/*intl*lI genes. No class 3 integrons were detected.

**Discussion:** In this study, multidrug resistance was seen in *Shigella* isolates similar to that in isolates from other geographical areas. This is possible due to inappropriate use of antimicrobials. Furthermore, prevalence of multidrug resistance was significantly linked to the presence of integrin genes.

**Conclusion:** A class 2 integron plays a role in presence of multidrug resistance in *Shigella* spp. It is vital to prevent the spread of antibiotic resistance through continuous monitoring.

## Zusammenfassung

**Hintergrund:** Die Zunahme multiresistenter *Shigella* spp. ist ein globales Gesundheitsproblem wachsender Bedeutung. Integrons sind Genexpressionssysteme, die von Integrase-Genen gesteuert werden. Zielsetzung der Studie war die Detektion von Klasse 1, 2 und 3 Integrons und die Bestimmung der antimikrobiellen Resistenz von *Shigella* spp., die von pädiatrischen Patienten mit Diarrhoe isoliert wurden.

**Material und Methoden: **Von Januar bis Dezember 2015 wurden 16 *Shigella* spp. aus 310 nicht-duplikativen Durchfall-Stuhlproben im Children’s Medical Center, Tehran, gemäß Guideline des Clinical and Laboratory Standards Institute isoliert. Zur Amplifikation der I, II und III Integron-assoziierten Integrase(*intl*)-Gene wurde die Multiplex PCR eingesetzt.

**Ergebnisse:** In 16 (5,2%) der 310 Stuhlproben wurden 7-mal *S. sonnei* und 9-mal *S. flexneri* isoliert. 6,2%, 50%, 31,2%, 6,2%, 81,2%, 56,2% bzw. 31,2% der Isolate waren resistent gegen Gentamicin, Chloramphenicol, Nalidixinsäure, Ciprofloxacin, Tetracycline, Ampicillin und Trimethoprim-Sulfamethoxazol. Mittels Multiplex PCR wurde nachgewiesen, dass 6,2% (1/16), 31,2% (5/16), 50% (8/16) der *Shigella* Isolate *intl*I, *intl*II bzw. beide Gene trugen. Klasse III Integrons wurden nicht detektiert.

**Diskussion:** Bei *Shigella*-Isolaten wurde ähnlich zu anderen geographischen Regionen Multiresistenz nachgewiesen. Das wird begünstigt durch nicht Leitlinien gerechten Einsatz von Antibiotika. Die Prävalenz der Multiresistenz war signifikant mit dem Vorhandensein von Integrin-Genen assoziiert.

**Schlussfolgerung:** Das Klasse 2 Integron ist von Bedeutung für die Multiresistenz von *Shigella* spp. Es ist wichtig, die Ausbreitung von Antibiotikaresistenzen durch kontinuierliche Überwachung zu verhindern.

## Introduction

Dysentery caused by *Shigella* spp. is a major public concern worldwide and is responsible for approximately 5 to 10% of diarrheal diseases in many areas [1]. Recently in Asia, the number of dysentery cases was estimated at nearly 91 million, resulting in 414,000 deaths each year. In general, *Shigella* spp. are categorized into four serogroups, including *S. dysenteriae*, *S. flexneri*, *S. boydii* and *S. sonnei* [2]. Of these serogroups, *S. flexneri* is the most common, followed by *S. sonnei*. However, dysentery caused by *Shigella* spp. is usually self-limited, and antibiotic therapy is mostly effective not only in treating the dysenteric infection, but also in decreasing the duration of the disease and fecal shedding of the pathogen [3]. Over the last decades, *Shigella* spp. have increasingly acquired resistance to various antimicrobials, including ampicillin, streptomycin, tetracycline and trimethoprim-sulfamethoxazole. The antibiotic resistance phenomenon in *Shigella* spp. commonly occurs due to mobile genetic elements (MGEs) such as R plasmids, transposons and integrons. Mobile genetic elements can mediate the distribution of resistance factors among the bacterial species, even genera. Furthermore, integrons with resistance gene cassettes have been recognized in MGEs. Resistance to antimicrobials in *Shigella* spp. is sometimes associated with class 1 and class 2 integrons, which comprise resistance gene cassettes. There are two types of class 1 integrons found in *Shigella* plasmids or chromosomes: atypical and classical integrons. These integrons are linked to gene cassettes of trimethoprim (*dfr*A1), esterase/lipase (*est*X), streptomycin (*aad*A1) and ampicillin (*bla*_oxa30_). Class 2 integrons carrying Tn7 are frequently present in *S. sonnei* and their gene cassettes contain *dfr*A1, streptothricin-acetyl-transferase gene (*sat*-1) and *aad*A1 [4], [5], [6], [7]. The aims of the current study were molecular analysis of integrons and antimicrobial resistance profiling in *Shigella* spp. isolated from acute pediatric diarrhea patients at the Children’s Medical Center, Tehran, Iran.

## Materials and methods

### Bacterial isolation

In a cross-sectional study, 310 non-duplicative and non-reiterative diarrheal stool samples were collected from children admitted to the Children’s Medical Center in Tehran, Iran, from January to December 2015. Samples were transferred to the laboratory in Cary-Blair media (Merck, Germany). Samples were cultured, and the bacteria isolated and identified using conventional biochemical as well as microbiological methods in addition to the API-20E system (BioMerieux, France). *Shigella* polyvalent agglutinating antisera were purchased from MAST, UK.

### Antimicrobial susceptibility test

Antimicrobial susceptibility testing was carried out using Mueller-Hinton agar plates (Merck, Germany) and the Kirby-Bauer method as recommended by the Clinical and Laboratory Standards Institute (CLSI document: M100-S14). The antimicrobial agents included gentamicin (GEN 10 µg), chloramphenicol (CHL 30 µg), streptomycin (STR 10 µg), nalidixic acid (NA 30 µg), ciprofloxacin (CIP 5 µg), tetracycline (TET 30 µg), ampicillin (AMP 20 µg) and trimethoprim-sulfamethoxazole (SMZ-TMP 5 µg) (MAST, UK). *Shigella flexneri* ATCC 12022 and *S. sonnei* ATCC 9290 were used as positive and *Escherichia coli* ATCC 25922 and* Pseudomonas aeruginosa* ATCC 27853 as negative controls.

### Integron gene detection

Multiplex PCR (M-PCR) for detection of *intl*I, *intl*II and *intl*III genes was carried out using a Master Cycler gradient PCR machine (Eppendorf, Germany). Microbial DNA was extracted using the boiling method from the colonies grown overnight on xylose lysine deoxycholate (XLD) agar. The primer sequences used in M-PCR are described in Table 1 (Tab. 1). The PCR reaction mixture was prepared in a total volume of 20 µl, consisting of 1 µl of template DNA, 2 µl of 10x PCR buffer, 0.6 µl of 50 mM MgCl_2_, 0.6 µl of 10 mM dNTPs, 0.5 µl of each primer, 0.7 µl of 5 U/µl Taq DNA polymerase (Amplicon, Denmark) and 12.1 µl of double-distilled water. The reaction mixture was transferred to a gradient thermal cycler (Eppendorf, Germany) with the following cycling program: initial denaturation at 94°C for 2 min followed by 33 cycles; each cycle included denaturation at 94°C for 30 s, annealing at 56°C for 30 s and elongation at 72°C for 30 s. Final elongation was carried out at 72°C for 10 min. Amplified products were visualized by electrophoresis in 1.5% agarose gels and staining with ethidium bromide. 

### Statistical analysis

Correlation between the occurrence of *intl*I, *intl*II and *intl*III genes and multidrug resistance was calculated using Fisher’s exact test. A *P*-value <0.05 was considered statistically significant. 

## Results

### Bacterial isolation

Of 310 stool samples, 16 (5.2%) samples were positive for *Shigella* spp. Of these 16 positive samples, 7 (43.7%) and 9 (56.3%) samples were identified as *S. sonnei* and *S. flexneri*, respectively. The mean age of the patients was six years, with 165 (53.2%) boys and 145 (46.7%) girls participating in the study. Nine (56.2%) bacterial species were isolated from children at ages 1 month to 2 years, and 7 (43.7%) in ages ranged from 2 to 12 years. 

### Antimicrobial susceptibility test

The results showed that 6.2%, 50%, 31.2%, 6.2%, 81.2%, 56.2% and 31.2% of bacterial isolates were resistant to gentamicin, chloramphenicol, nalidixic acid, ciprofloxacin, tetracycline, ampicillin and trimethoprim-sulfamethoxazole, respectively (Table 2 (Tab. 2)). All isolates were resistant to streptomycin. *S. flexneri* isolates showed high levels of resistance to streptomycin (100%), tetracycline (85.7%), ampicillin (85.7%) and chloramphenicol (71.4%), while low-level resistance was detected to ciprofloxacin (14.3%) and gentamicin (14.3%). Furthermore, 100%, 77.7%, 33.3%, 22.2% and 11.1% of *S. sonnei* isolates were resistant to streptomycin, tetracycline, ampicillin/chloramphenicol, nalidixic acid and trimethoprim-sulfamethoxazole, respectively. All *S. sonnei* isolates were fully susceptible to gentamicin and ciprofloxacin. Moreover, 55.1% (n=4/7) of *S. flexneri* and 33.3% (n=3/9) of *S. sonnei* isolates were resistant to streptomycin, tetracycline and ampicillin (Table 2 (Tab. 2)).

### Integron gene detection

**Figure 1:**
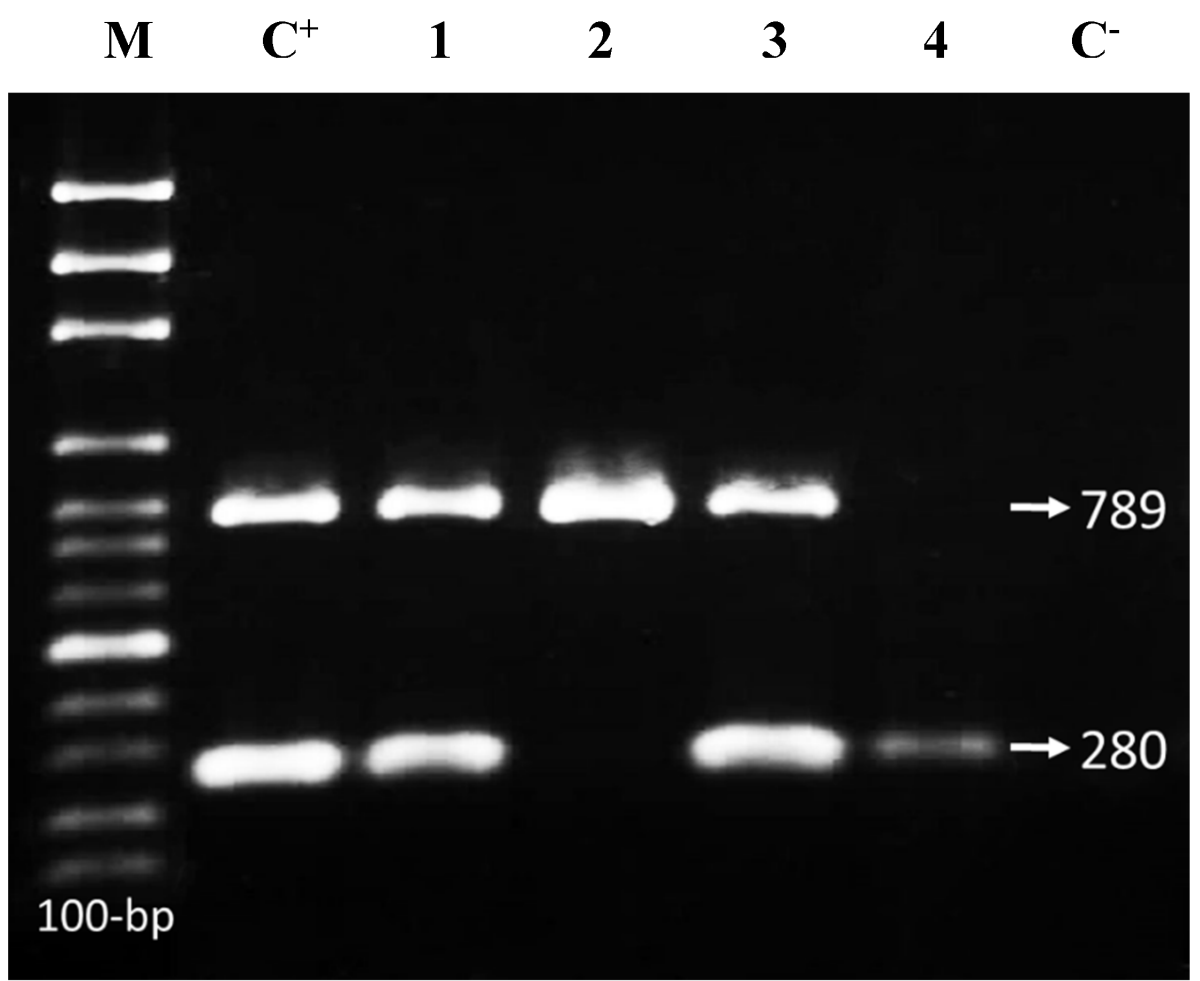
M-PCR products of *intl*I/II genes (280/789 bp). M: 100-bp DNA ladder; C+, positive control (*S. flexneri* ATCC 12022/*S. sonnei* ATCC 9290), Lanes 1 & 2: *S. flexneri*; Lanes 3 & 4: *S. sonnei* strains; C–, negative control (*E. coli* ATCC 25922)

Totally, 6.2% (1/16), 31.2% (5/16) and 50% (8/16) of the *Shigella* isolates carried *intl*I, *intl*II and both *intl*I/*intl*II genes, respectively (Figure 1 (Fig. 1)). No class III integrons were detected. The prevalence of *intl*II was significantly higher than that of *intl*I and in multidrug resistant (MDR) isolates than in isolates with resistance to two or fewer two drugs (P<0.05). Furthermore, 12.5% (n=2/16) of the isolates were negative for *intl*I, *intl*II and *intl*III genes (Table 3 (Tab. 3)).

### Statistical analysis

No significant difference was seen between the *intl*I gene and MDR (P>0.05). The correlation between the presence of *intl*II or *intl*I/II genes and antibiotic resistance was statistically significant (Table 4 (Tab. 4)).

## Discussion

Increased resistance of *Shigella* spp. to many antimicrobial agents presents a major threat to public health. Over the past decades, excessive use of antimicrobials and vast horizontal gene transfer have led *Shigella* spp. to become resistant to most routinely used antimicrobials. Primarily, tetracycline and sulfonamides were effective in the treatment of shigellosis, but the bacterial strains quickly established resistance to these agents. Later, ampicillin and trimethoprim-sulfamethoxazole were used to treat shigellosis. Antimicrobial resistance is common in *Shigella* spp., mostly to tetracycline, trimethoprim-sulfamethoxazole and other sulfonamides. Increased bacterial resistance to ampicillin, chloramphenicol and trimethoprim-sulfamethoxazole is a serious threat. These are low-cost antimicrobials used widely for the treatment of shigellosis [8], [9], [10]. In general, multidrug-resistant *Shigella* spp. have been reported from Africa, Europe, Asia and South America. In the current study, all isolates were resistant to streptomycin. Thirteen (81.2%) and one (6.2%) *Shigella* isolates were resistant to tetracycline and gentamicin/ciprofloxacin, respectively. Similar results have been published from other studies in developing countries [11], [12], [13]. All isolates (100%) of *S. sonnei* were fully susceptible to gentamicin and ciprofloxacin. Moreover, 77.7% (n=7/9) and 11.1% (n=1/9) of *S. sonnei* isolates were resistant to tetracycline and trimethoprim-sulfamethoxazole, respectively. The results showed that the highest and lowest resistance to ampicillin and gentamicin/ciprofloxacin in *S. flexneri* were 85.7% (n=6/7) and 14.3% (n=1/7), respectively. Pourakbari et al. reported that *S. flexneri* was more multiresistant than other species [14]. Results by Zhu et al. [15] showed that resistance of *S. sonnei* to ampicillin and ciprofloxacin varied and was relatively infrequent, while antimicrobial resistance was common in *S. flexneri*. Later, Shen et al. [16] demonstrated that resistance of *S. flexneri* (serotypes 1a) to antimicrobials was significantly higher, including 88.0%, 89.2%, 85.5% and 79.5%, to ampicillin, nalidixic acid, tetracycline and trimethoprim-sulfamethoxazole, respectively. In a similar study by Jafari et al. [17], most *Shigella* isolates were reported to be resistant to tetracycline (95%) and trimethoprim-sulfamethoxazole (91.7%). The maximum resistance (60.2%) was observed in *S. sonnei*. In the present study, multidrug resistance was detected in 57.1% (n=4/7) of *S. flexneri* and 33.3% (n=3/9) of *S. sonnei* isolates. This was in contrast to the results of the studies by Zhu et al. [15] and Jafari et al. [17]. This conflict may be due to geographical distribution, source of samples and level of hygiene.

Of the three classes of integrons linked to antimicrobial resistance, the class I integron is the most frequently found in clinical isolates of Gram-negative bacteria [18]. The class II integron is the most predominant integron in *S. sonnei* [5]. In the current study, 6.2% (n=1/16), 31.2% (n=5/16), 0% (n=0/16) and 50% (n=8/16) of *Shigella* isolates carried *intl*I, *intl*II, *intl*III and both *intl*I/*intl*II genes, respectively. These results are similar to those of Shen et al. [16], Ranjbar et al. [19] and Nógrády et al. [20]. The present study has clearly shown that the prevalence of *intl*II is noticeably greater than that of *intl*I. Furthermore, the prevalence rate of these genes in MDR isolates with resistance to ≥3 drugs is higher than that in MDR [6], [15]. Zhu et al. [15] described that *Shigella* spp. included a high frequency of MDR and a high occurrence of classes I and II integrons at the same time; the prevalence of the *intl*II gene was significantly associated with MDR isolates (P<0.05) [21]. Currently, the presence of class II integrons and rate of MDR are linked in *Shigella* spp.; therefore, class II integrons may play a role in the presence of MDR in *Shigella* spp. This suggests a gene linkage between class II integrons and other antimicrobially resistant genes. Furthermore, this suggests that class II integrons work together with other determinants of genetic resistance. Further studies are needed to confirm these possibilities. The possible link of class II integrons with other antimicrobial resistance genes would help to employ class II integrons as molecular biomarkers to screen MDR in *Shigella* spp.

## Conclusion

Antimicrobial resistance of *Shigella* spp. in developed countries appears to be frequent, and associated with their epidemiology in developing countries. Mostly, *Shigella* strains that carry class I or II integrons show emergence of MDR. Preventing the distribution of antibiotic resistance and spread of integrons is a matter of general urgency. Therefore, continuous monitoring schemes must be implemented to prevent further spread of MDR *Shigella* spp.

## Notes

### Competing interests

The authors declare that they have no competing interests.

### Acknowledgement

This work was supported by a Vice-Chancellor for Research grant (No. 23125), Tehran University of Medical Sciences, Tehran, Iran. We thank the Children’s Medical Center in Tehran for providing isolates and epidemiological and demographic data.

## Figures and Tables

**Table 1:**
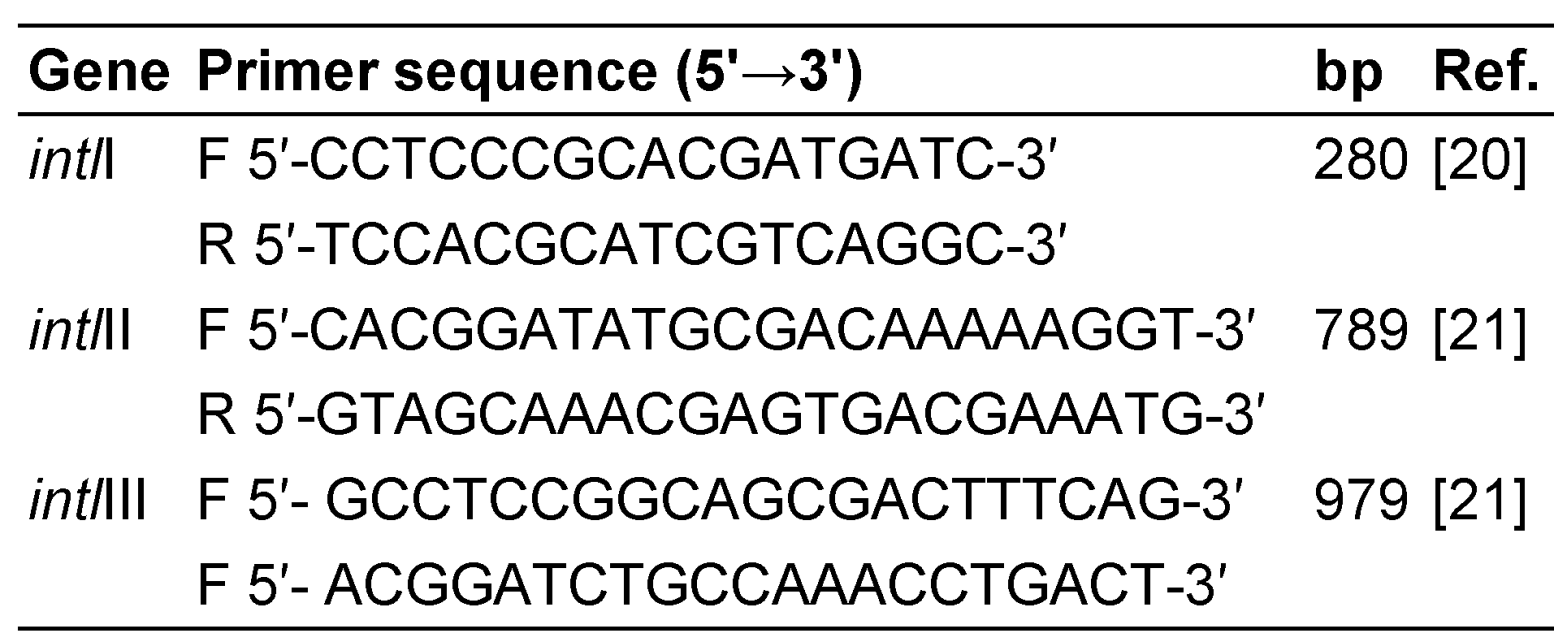
PCR primers used in this study

**Table 2:**
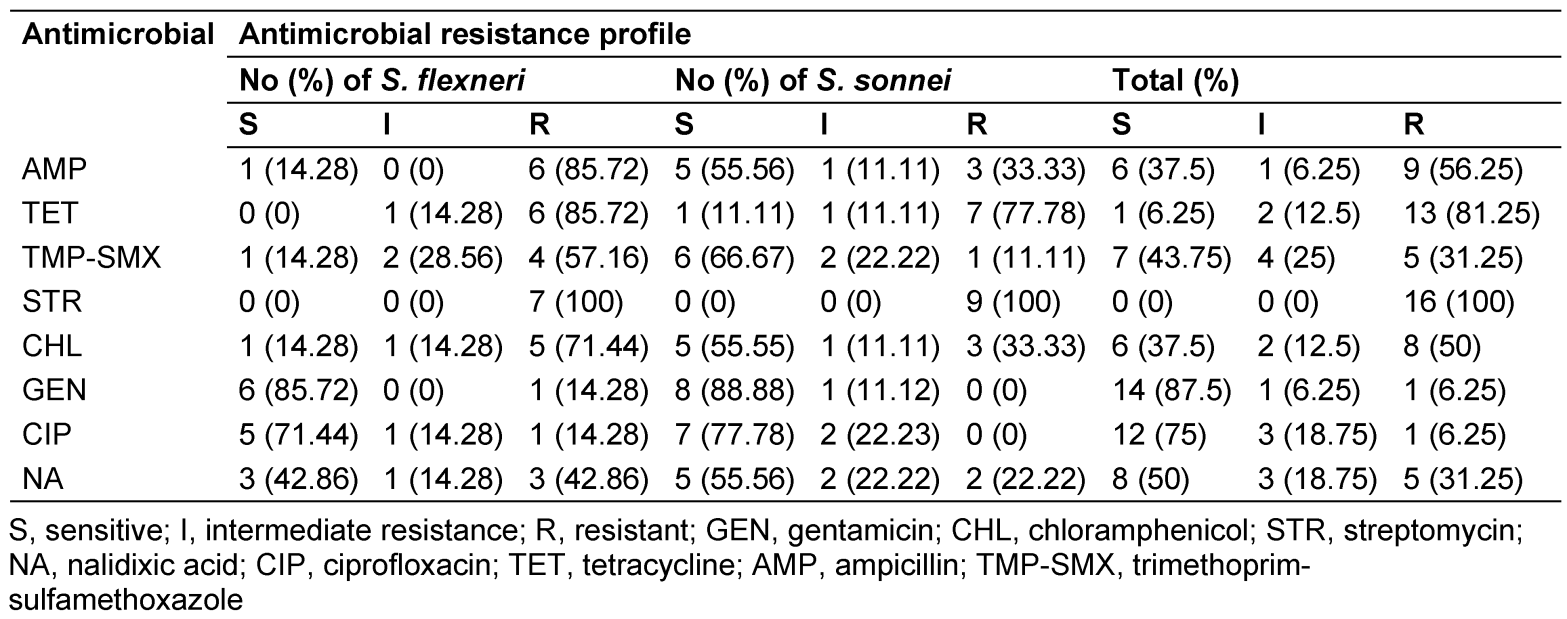
Antimicrobial susceptibility of the *Shigella* isolates

**Table 3:**
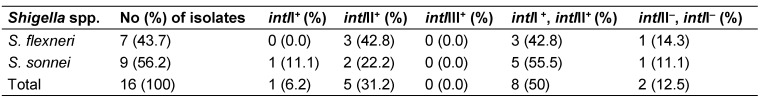
Distribution of class I, II and III integrons in *Shigella* spp.

**Table 4:**
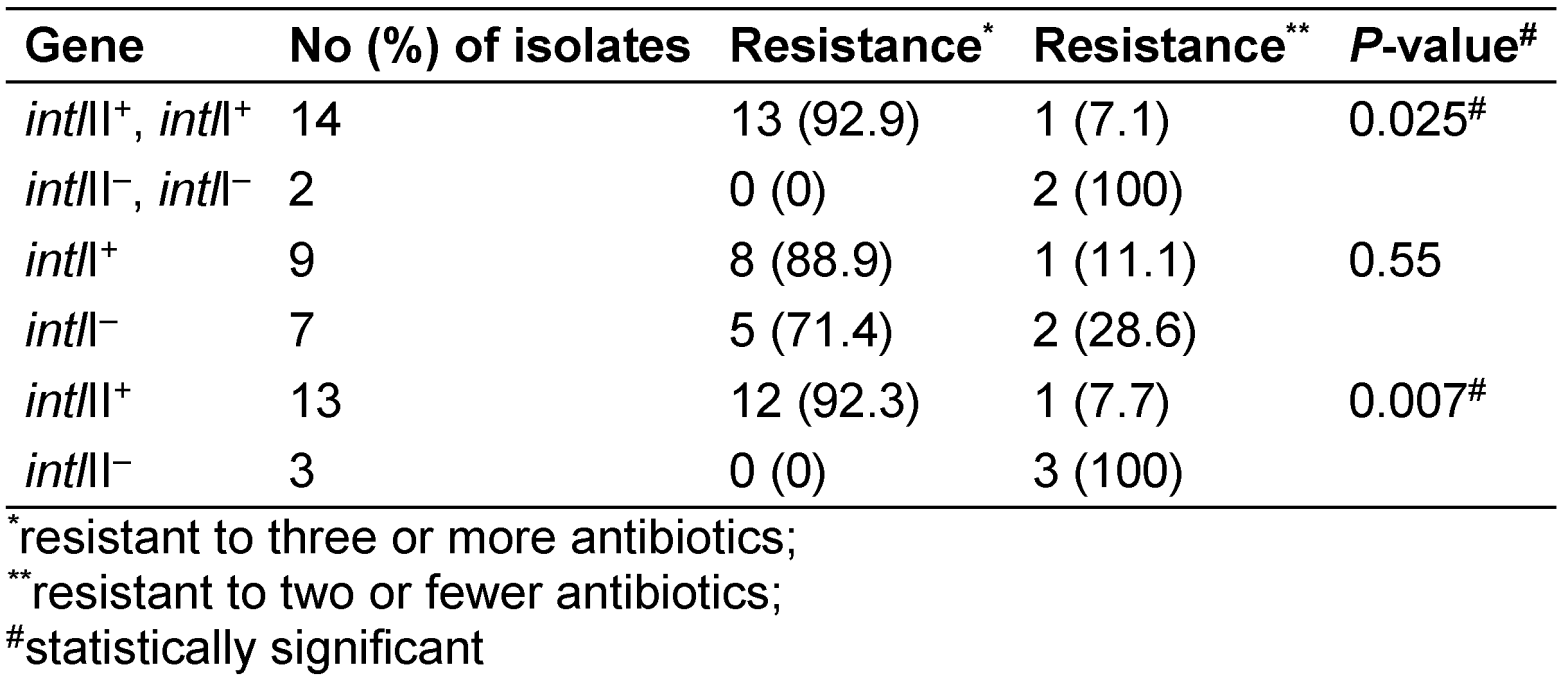
Integrons and multiresistance in the *Shigella* isolates
